# Anatomofunctional Zone-Based Marking of the Lower Limbs (Angioderma Leg Mapping): A Proposed Standardized Methodology for Therapeutic Planning

**DOI:** 10.7759/cureus.116127

**Published:** 2026-09-12

**Authors:** Eduardo Zeilmann, Brenno Augusto S Mello Netto, Gilmar S Santos

**Affiliations:** 1 Vascular Surgery, Instituto de Flebologia Moderna (IFE), Itajaí, BRA; 2 Vascular Surgery, Seabra Excelencia Vascular, Vitória, BRA; 3 Vascular Surgery, Vein Clinic, Salvador, BRA

**Keywords:** body contouring, lipedema, liposuction, lower limb, lymphatic system, surface anatomy, zones of adherence

## Abstract

The aesthetic-functional planning of the lower limbs is frequently conducted empirically, using idiosyncratic markings and limited standardization among specialists who work in this field. The absence of a common topographic language restricts clinical communication, technical reproducibility, documentation of results, and the integration between the aesthetic approach and the vascular/lymphatic assessment of the segment. This is a descriptive study of methodological development. Based on a review of the anatomic and surgical literature and prospective clinical application during body-contour consultations, six topographic zones of the lower limbs were defined: gluteal, trochanteric (saddlebag), thigh (anterior, medial, and posterior subunits), knee, crural (calf), and talocrural (ankle), each delimited by reproducible surface bony and muscular landmarks. Onto this map, the cartography of the zones of adherence, superficial and deep adipose planes separated by the superficial fascia, saphenous venous axes, and main lymphatic collector pathways was overlaid. A four-stage marking protocol was defined (orthostatism, marking of bony landmarks, zone delimitation, and coding of plane and intensity), with standardized photographic documentation. For each zone, the following are presented: anatomic boundaries, muscular and adipose content, vascular and lymphatic correlates, zones of adherence to be preserved, recommended working plane (deep, intermediate, or superficial), specific risks, and expected aesthetic outcomes. The map provides a common nomenclature among surgeons and enables technology to be prescribed by anatomic zone rather than by generic "region". This anatomofunctional zone-based marking provides a simple, low-cost, and reproducible instrument to structure the therapeutic planning of the lower limbs, aligning the aesthetic objective with the anatomic, vascular, and lymphatic constraints of the segment. Interobserver reliability studies and comparative clinical outcome studies are the necessary next step toward validation.

## Introduction

The contour of the lower limbs is one of the most demanding domains of body-contour surgery and aesthetic medicine. Unlike the abdomen, where the response to treatment is relatively predictable, the lower limbs combine three factors that increase the complexity of planning: (a) a stratified and regionally heterogeneous adipose architecture; (b) the presence of dermofascial zones of adherence whose violation produces contour deformities that are difficult to correct; and (c) a dense superficial venous and subcutaneous lymphatic network whose integrity determines both safety and the medium-term aesthetic outcome.

Since the systematization of suction-assisted lipolysis by Illouz and the introduction of the tumescent technique by Klein, liposuction has consolidated itself as a safe, high-demand procedure [1,2]. Subsequent refinements such as superficial and subdermal liposuction for skin retraction [3], ultrasound-assisted high-definition liposculpture [4], and laser-assisted lipolysis [5] have broadened the technical repertoire but have shifted the bottleneck of the result from "how to aspirate" to "where, at which plane, and at what intensity." This shift makes preoperative topographic planning an independent determinant of outcome.

The anatomic basis for such planning is well established. Markman and Barton demonstrated that the subcutaneous tissue of the trunk and lower limbs is organized into a superficial areolar adipose layer and a deep lamellar adipose layer, separated by a membranous superficial fascia [6]; this organization was subsequently formalized by Lockwood as the superficial fascial system [7]; recent anatomic studies have confirmed and detailed this trilaminar organization in the thigh [8]. Rohrich et al. described the zones of adherence, that is, regions in which the superficial fascial system attaches densely to the deep fascia, and emphasized that their preservation is essential to avoid postoperative irregularities and depressions [9]. In parallel, the venous nomenclature of the lower limbs was standardized by international consensus, defining the saphenous compartment and its fascial boundaries [10], and the CEAP classification provides a common language for chronic venous disease [11]. In the lymphatic territory, recent mapping evidence demonstrates that the main collectors of the lower limb follow predictable courses and can be spared when mapped [12], and reviews of tumescent liposuction suggest that the technique, when properly performed, does not cause clinically relevant lymphatic injury [13].

Despite this density of knowledge, everyday clinical practice still operates with a largely idiosyncratic preoperative marking: each operator draws his or her own map, with proprietary nomenclature and without explicit correspondence between the skin tracing and the underlying structures. The consequences are practical and measurable: difficulty in communication between the surgical team and the venous-lymphatic rehabilitation team, the impossibility of comparing results between operators or between sessions, non-standardized photographic documentation, and prescription of technologies by generic regions ("thigh," "leg") rather than by anatomofunctional unit.

The present study proposes a response to this methodological gap. We describe a methodology for anatomofunctional marking of the lower limbs that segments the limb into six topographic zones defined by palpable bony and muscular landmarks and overlays onto this segmentation three clinically decisive information layers: the adipose planes, the zones of adherence, and the vasculolymphatic axes. The objective is to offer a reproducible planning instrument rather than a new operative technique, capable of integrating, within a single document, the patient's aesthetic goal and the anatomic constraints of the segment.

## Technical report

Study design

The descriptive methodological development study was conducted in two phases: (i) a structured review of the anatomic and surgical literature and (ii) construction of the zone map and marking protocol.

Literature search

A search was conducted in the MEDLINE/PubMed (U.S. National Library of Medicine (NLM), Bethesda, Maryland), Embase (Elsevier, Amsterdam, the Netherlands), SciELO (Scientific Electronic Library Online, São Paulo, Brazil), and Cochrane Library (Wiley, Hoboken, New Jersey), covering the period from (month/year) to (month/year), with no initial date restriction. The following free-text descriptors were used: lipectomy, liposuction, body contouring, lower extremity, subcutaneous fat, superficial fascia, zones of adherence, lymphatic vessels, saphenous vein, lipedema, and cellulite. Anatomic studies, surgical series, systematic reviews, consensus statements, and guidelines in Portuguese, English, or Spanish were included.

Zone-map construction

Each zone was defined by a triple criterion, simultaneously requiring: (1) Delimitation, the borders must be traceable on the skin using bony or muscular landmarks palpable in orthostatism, without imaging. (2) Functional coherence, the zone must correspond to a homogeneous adipose, vascular, or biomechanical behavior. (3) Decision relevance, the distinction between the zone and its neighbors must change management (aspiration plane, technology used, intensity, or contraindication).

Zones that failed to satisfy all three criteria were merged with adjacent ones or treated as subunits.

Nomenclature. All zones are named according to the *Terminologia Anatomica* (regio glutealis, regio femoris anterior/medialis/posterior, regio genus, regio cruris, and regio talocruralis), with the corresponding lay term in parentheses at first mention only; "saddlebag" (culote) is treated as the lay synonym for the trochanteric/lateral thigh adipose deposit. Venous terminology follows the international consensus on the nomenclature of the veins of the lower limbs [10,11].

Marking protocol

Step 1: Positioning and Inspection

The patient is placed in orthostatism, barefoot, with the feet shoulder-width apart and the upper limbs abducted, under frontal and tangential lighting. Axis asymmetry, valgus and varus deformities, competence of the plantar arch, and the presence of tissue ptosis are recorded.

Step 2: Landmark Marking

The following are marked: iliac crest, anterosuperior and posterosuperior iliac spines, greater trochanter, medial and lateral femoral condyles, tibial tuberosity, and medial and lateral malleoli. These landmarks form the skeleton of the map and ensure its reproducibility between sessions and operators.

Sartorius marking technique: With the patient in orthostatism, hip flexion, abduction, and lateral rotation are requested, together with knee flexion against mild resistance, the movement of "crossing the legs while seated," which is the characteristic action of this muscle. Under this contraction, the sartorius belly emerges as an oblique cord. Its anterior border is marked in blue, from its origin at the ASIS to the medial femoral condyle.

Significance for the method: The anterior border of the sartorius is the functional dividing line between the anterior and the medial femoral subunits, the two subunits in the entire map that require the most divergent management. Anterior to this line, the subcutaneous tissue is thinner, more adherent, and more tolerant of aspiration; posterior and medial to it, it is looser, with thinner skin, a higher prevalence of laxity, and the presence of the lymphatic collectors accompanying the great saphenous vein. Tracing this line converts a conceptual distinction into a visible boundary on the skin.

The gluteal crease is not the inferior border of the gluteus maximus, a frequent misconception. It is a dermofascial structure: a condensation of the superficial fascial system that anchors the dermis to the deep fascia, supporting the inferior pole of the buttock against gravity. Cadaveric dissections have demonstrated that the superficial fascial system in the region of the gluteal crease exhibits a clearly defined pattern of regional variation, with a dense and resistant medial component that functions as a containment network, preventing inferior migration of the deep gluteal fat toward the thigh and maintaining the load equilibrium of the region. Immediately above and deep to this condensation lies the infragluteal fat pad, a deep deposit whose integrity is a prerequisite for projection of the inferior pole. A recent cadaveric and radiographic study specifically reassessed the fascial anchoring structures of the infragluteal crease, providing the direct anatomic basis for treating it as a supporting structure rather than as a simple cutaneous fold.

Gastrocnemius marking: With the patient in orthostatism, rising onto the forefoot (active plantar flexion against body weight), and during this contraction, the following are marked in blue: (a) the contour of each gastrocnemius belly, medial and lateral, separately; (b) the myotendinous junction, the point at which the muscular bellies end and the aponeurosis organizes into the Achilles tendon; and (c) the proximal limit of the soleus, when visible lateral to the gastrocnemius (Figure 1).

**Figure 1:**
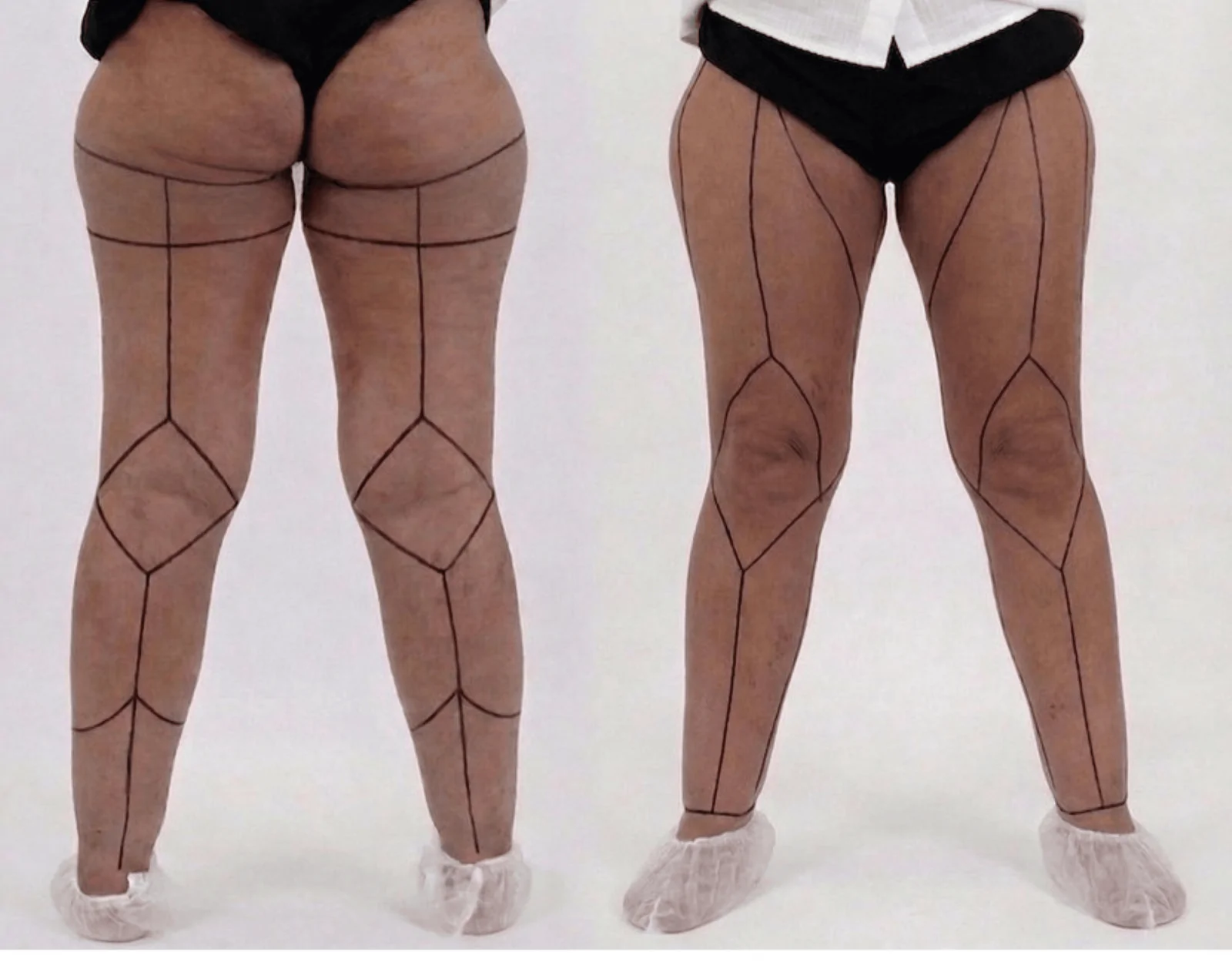
Anatomical-functional marking of lower limb regions: posterior and anterior views.

Step 3: Zone Delimitation

The six zones described are traced in distinct colors, respecting the landmarks from Step 2. The zones of adherence are cross-hatched in red as territories of exclusion or minimal intervention. In a fourth color (purple), the neurovasculolymphatic risk corridors are traced: the great saphenous vein and the accompanying lymphatic collectors on the medial aspect of the thigh and leg [14-16], the small saphenous vein along the posterior midline of the leg, the popliteal fossa, the distal saphenous nerve [17], and the sural nerve. This chromatic layer distinguishes the anatomofunctional vascular marking from a conventional aesthetic map.

Complementary clinical assessment per zone

Marking is always accompanied by (a) a two-finger pinch test to estimate the thickness of the superficial and deep layers; (b) a dynamic pinch test to grade skin laxity; (c) cellulite grading using the validated photonumeric scale of Hexsel, Dal'Forno, and Hexsel [17]; (d) lipedema screening according to international consensus criteria and the S2k guideline [18]; (e) clinical venous assessment with the CEAP classification [12], supplemented by color Doppler ultrasound in cases with CEAP ≥ C2, a history of thrombosis, or unexplained volumetric asymmetry; and (f) perimetry at fixed points referenced to bony landmarks (10 and 20 cm above the base of the patella; 15 cm below the base of the patella; maximal calf circumference; and 5 cm above the medial malleolus) (Figure 2).

**Figure 2:**
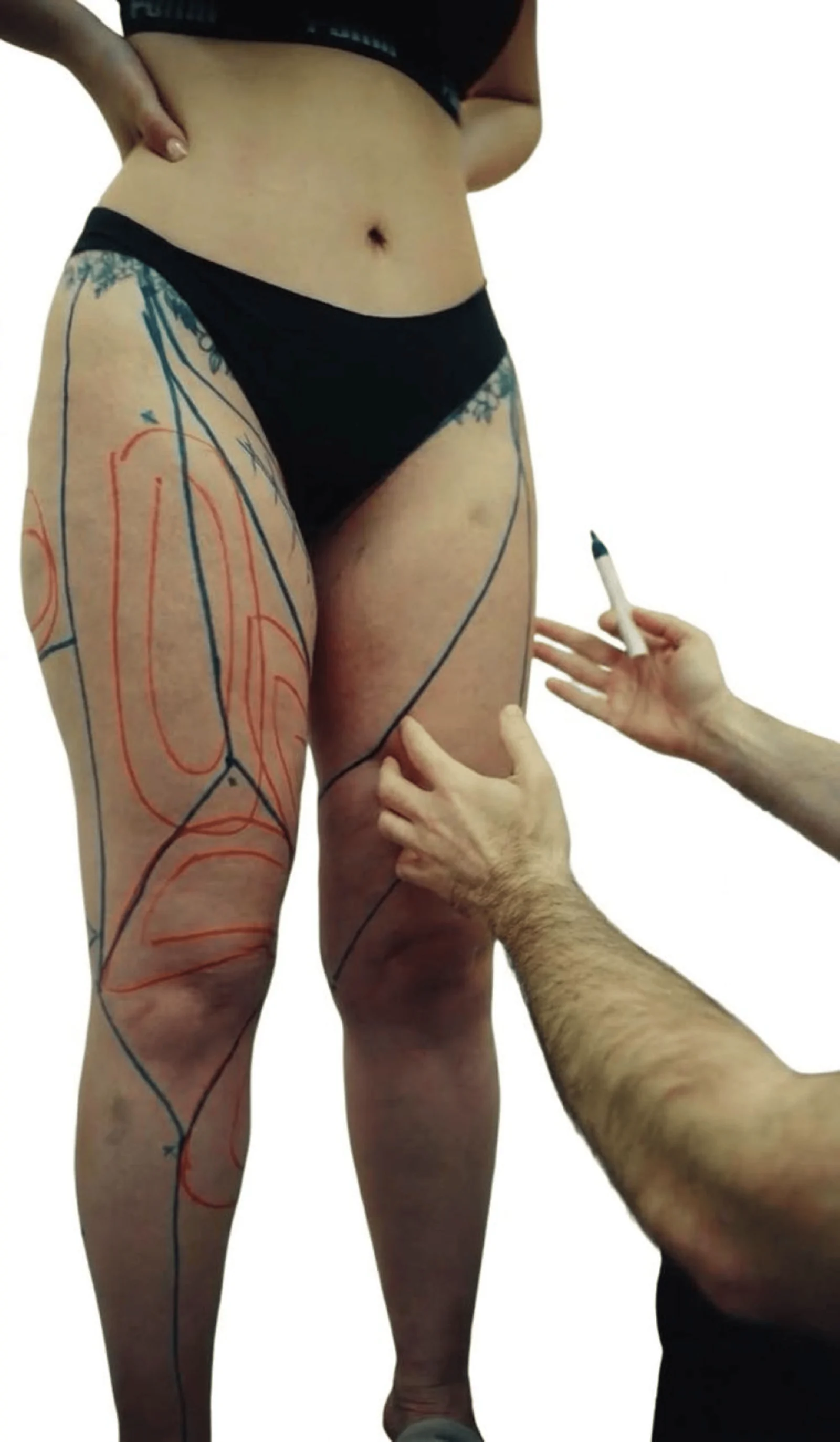
Practical application of leg mapping using the Angioderma Leg Mapping method.

Results: Description of the zones

Common Stratigraphic Basis

In all zones, the subcutaneous tissue is treated as three functionally distinct strata [7,8,19]: the superficial (areolar) layer, small lobules, dense vertical septa, and an intimate relationship with the dermis [20-23]. This is the stratum responsible for surface texture and for the appearance of cellulite [24-26], whose origin lies in the orientation of the fibrous septa and in dermal lobular herniation. Aspiration in this plane produces skin retraction, but within a narrow margin of error and with a risk of irregularity and adherence [27-29]. The superficial (membranous) fascia, with the natural cleavage plane and the principal safety reference. The deep (lamellar) layer, with larger lobules having loose, oblique septa. This is the volume stratum and the primary target of reduction liposuction, and it offers the widest safety margin.

The derived operational rule is uniform: volume comes from the deep plane; shape and retraction come from the superficial plane; safety comes from respecting the superficial fascia and the zones of adherence.

Zone I: Gluteal Region

Boundaries: superiorly, the iliac crest; inferiorly, the gluteal crease; medially, the intergluteal sulcus; laterally, a vertical line through the greater trochanter. Content: gluteus maximus and medius; a thick adipose compartment, predominantly superficial, with high septal density. Zones of adherence to preserve: gluteal crease and lateral gluteal depression [9]. Functional correlate: the unit that determines projection and the lumbogluteal transition; its morphology accounts for a large share of the aesthetic perception of the posterior segment [22,23]. Derived management: as a rule, the zone is a restricted donor or recipient zone. Aggressive aspiration of the inferior pole deepens the gluteal crease and produces iatrogenic ptosis. Definition is achieved by treating the boundary zones (sacral, flank, and trochanteric), not by central emptying.

Zone II: Trochanteric Region (Trochanteric Deposit; Lay Synonym "Saddlebag")

Boundaries: superiorly, the line between the ASIS and the iliac crest; centrally, the greater trochanter; inferiorly, the transition to the proximal third of the lateral thigh. Content: deep, hormone-dependent adipose deposit, overlying the iliotibial tract. Zones of adherence to preserve: the distal lateral thigh over the iliotibial tract and the lateral gluteal depression [9]. Derived management: a classic, high-yield target in the deep plane; superficial aspiration in this zone is the most frequent cause of visible lateral irregularity under oblique lighting. The characteristic technical error is aspiration that crosses the inferior border and invades the zone of adherence of the iliotibial tract, creating a difficult-to-correct "step" depression.

Zone III: Anterior, Medial, and Posterior (Thigh)

III-a: Anterior. Over the quadriceps, a relatively thin and uniform adipose layer. It tolerates conservative aspiration in the deep plane; the marking must preserve the projection of the rectus femoris, whose obliteration produces a flattened, unnatural appearance.

III-b: Medial. A loose adipose layer, thin skin, low septal density, and high prevalence of laxity. A critical territory: it contains the course of the great saphenous vein within the saphenous compartment [10] and the superficial medial lymphatic collectors that ascend in parallel toward the inguinal lymph nodes [13]. It is the subunit with the greatest risk of irregularity, residual laxity, and lymphatic compromise. Management: conservative aspiration, predominantly deep, with fine cannulas; preferential indication of technologies with a skin-retraction component (UAL, LAL, and RF) over volumetric aspiration alone [5,6]; explicit marking of the saphenous axis as a caution corridor.

III-c: Posterior. Over the hamstrings. Zones of adherence to preserve: distal posterior thigh and middle medial thigh [9]. Distal posterior aspiration is the most direct route to the "banana-roll" deformity below the gluteal crease; the marking must leave this strip explicitly cross-hatched.

Zone IV: Genicular (Knee) Region

Boundaries: from 10 cm above the base of the patella to the tibial tuberosity; landmarks: femoral condyles, patella. Content: suprapatellar adipose compartment and, above all, the medial infrapatellar fat pad (the deposit most consistently responsible for loss of the leg line). Functional correlate: zone of biomechanical transition and lymphatic convergence; marking must respect the popliteal region, where the lymphatic collectors and the small saphenous vein converge. Derived management: small-volume aspiration, with fine cannulas, in the deep plane of the medial fat pad, performed with the knee in slight flexion. It is the zone with the highest aesthetic yield per milliliter aspirated from the entire limb, as small corrections produce a marked perception of slenderness while being simultaneously one of the most sensitive to prolonged edema.

Zone V: Leg/Calf Region

Boundaries: from the tibial tuberosity to the supramalleolar transition; landmarks: fibular head, tibial crest, gastrocnemius bellies, and Achilles tendon. Content: thin adipose compartment, distributed circumferentially; the medial aspect of the tibia is subcutaneous and represents a natural zone of adherence. Functional correlate: the seat of the calf-muscle pump, the central motor of venous return; the course of the small saphenous vein on the posterior surface and of the great saphenous vein on the medial surface [10]. Edema and retention in this zone frequently reflect venous or lymphatic disease rather than adipose excess, a distinction that the marking forces one to make explicit before any indication. Derived management: zone of restricted indication and outcome dependent on selection. A homogeneous, conservative, circumferential approach is recommended, exclusively in the deep plane, with rigorous and prolonged postoperative compression. It is the zone with the longest edema-resolution time in the entire limb. A clear relative contraindication exists in the presence of CEAP ≥ C3. It is also the zone with the highest reported rate of undesirable outcomes in specific calf and ankle series [24,25], which reinforces the requirement for rigorous selection and uniform technique [25].

Zone VI: Talocrural (Ankle) Region

Boundaries: from the malleoli to the transition with the dorsum of the foot. Content: minimal subcutaneous tissue; region of convergence of lymphatic collectors, tendons, and superficial neurovascular bundles. Derived management: zone of relative exclusion. Volume increase here is, in most cases, lymphatic or venous and not adipose. Its primary function in the map is diagnostic: the ankle is the sentinel of the segment; a positive Stemmer sign, persistent depressible edema, or supramalleolar asymmetry redirects the case from the aesthetic to the vascular/lymphatic pathway before any procedure is performed. When approached, it should be approached only with minimal technique, small-caliber cannulas, and prior mapping.

## Discussion

The method described here does not introduce a new surgical technique; it introduces a grammar of planning. Its value lies in three gains.

The first is reproducibility: By anchoring each boundary in a palpable bony landmark, the map can be redrawn by another professional or by the same professional at another session, with essentially the same tracing, which is a necessary condition for comparing outcomes and for conducting treatments in multiple stages.

The second is design-based safety: By cross-hatching the zones of adherence described by Rohrich et al. [9] before any aspiration decision, the method converts anatomic knowledge into an explicit graphic restriction. The same applies to the vasculolymphatic corridors: an axis drawn on the skin is an axis respected in the act.

The third is clinical interoperability: A zonal nomenclature allows surgeons to discuss the same case using the same vocabulary and enables technology prescription to shift from a regional to a zonal and planar basis. This is particularly relevant in populations in which the boundary between aesthetic and disease is thin.

Limitations

This is a descriptive methodological proposal that has not yet been formally validated. Inter-observer reliability of the tracing has not been quantified; to date, there is no comparison of aesthetic outcomes or of complication rates between cases planned with and without the method; delimitation by palpable landmarks has reduced accuracy in patients with elevated BMI; and the clinical-application sample is limited and single-center. Furthermore, the strength of the evidence supporting the superiority of specific technologies per zone is heterogeneous, with a predominance of case series [6].

## Conclusions

Anatomofunctional zone-based marking of the lower limbs organizes, in a single low-cost graphic instrument applicable in an outpatient setting, the surface topography, the adipose stratigraphy, the zones of adherence, and the venous and lymphatic axes of the segment. By replacing idiosyncratic marking with a nomenclature anchored to reproducible bony landmarks and with an explicit coding of plane, intensity, and technology, the method brings planning in vascular body-contour aesthetics and technology-assisted liposuction closer to the standards of reproducibility and documentation required by evidence-based medical practice. Its incorporation into routine practice is immediate; its formal validation, through reliability and outcome studies, is the necessary next step.
